# PIK3CA Genotype and a PIK3CA Mutation-Related Gene Signature and Response to Everolimus and Letrozole in Estrogen Receptor Positive Breast Cancer

**DOI:** 10.1371/journal.pone.0053292

**Published:** 2013-01-02

**Authors:** Sherene Loi, Stefan Michiels, Jose Baselga, John M. S. Bartlett, Sandeep K. Singhal, Vicky S. Sabine, Andrew H. Sims, Tarek Sahmoud, J. Michael Dixon, Martine J. Piccart, Christos Sotiriou

**Affiliations:** 1 Breast Cancer Translational Research Laboratory (BCTL), Jules Bordet Institute, Brussels, Belgium; 2 Division of Hematology and Oncology, Massachusetts General Hospital Cancer Center, Boston, Massachusetts, United States of America; 3 Endocrine Cancer Group, University of Edinburgh Cancer Research Centre, Western General Hospital, Edinburgh, United Kingdom; 4 Ontario Institute for Cancer Research, Toronto, Ontario, Canada; 5 Edinburgh Breast Unit, Institute of Genetics & Molecular Medicine, University of Edinburgh, Western General Hospital, Edinburgh, United Kingdom; 6 Novartis Pharma, East Hanover, New Jersey, United States of America; 7 Applied Bioinformatics of Cancer Group, Edinburgh Breakthrough Unit, University of Edinburgh Cancer Research Centre, Institute of Genetics & Molecular Medicine, Western General Hospital, Edinburgh, United Kingdom; 8 Department of Medicine, Jules Bordet Institute, Brussels, Belgium; University of Patras, Greece

## Abstract

The phosphatidylinositol 3′ kinase (PI3K) pathway is commonly activated in breast cancer and aberrations such as PI3K mutations are common. Recent exciting clinical trial results in advanced estrogen receptor-positive (ER) breast cancer support mTOR activation is a major means of estrogen-independent tumor growth. Hence the means to identify a responsive breast cancer population that would most benefit from these compounds in the adjuvant or earlier stage setting is of high interest. Here we study *PIK3CA* genotype as well as a previously reported PI3K/mTOR-pathway gene signature (*PIK3CA*-GS) and their ability to estimate the level of PI3K pathway activation in two clinical trials of newly diagnosed ER-positive breast cancer patients- a total of 81 patients- one of which was randomized between letrozole and placebo vs letrozole and everolimus. The main objectives were to correlate the baseline *PIK3CA* genotype and GS with the relative change from baseline to day 15 in Ki67 (which has been shown to be prognostic in breast cancer) and phosphorylated S6 (S240) immunohistochemistry (a substrate of mTOR). In the randomized dataset, the *PIK3CA*-GS could identify those patients with the largest relative decreases in Ki67 to letrozole/everolimus (R = −0.43, p = 0.008) compared with letrozole/placebo (R = 0.07, p = 0.58; interaction test p = 0.02). In a second dataset of pre-surgical everolimus alone, the *PIK3CA*-GS was not significantly correlated with relative change in Ki67 (R = −0.11, p = 0.37) but with relative change in phosphorlyated S6 (S240) (R = −0.46, p = 0.028). *PIK3CA* genotype was not significantly associated with any endpoint in either datasets. Our results suggest that the *PIK3CA*-GS has potential to identify those ER-positive BCs who may benefit from the addition of everolimus to letrozole. Further evaluation of the PIK3CA-GS as a predictive biomarker is warranted as it may facilitate better selection of responsive patient populations for mTOR inhibition in combination with letrozole.

## Introduction

The phosphatidylinositol 3-kinase (PI3K)/AKT/mammalian Target Of Rapamycin (mTOR) pathway is commonly deregulated in breast cancer and consequently, there is intense interest in the clinical development of agents that target this pathway [1]. The presence of genetic aberrations such as *PIK3CA* mutations, PTEN loss and HER2 amplification are frequent in breast cancer and whilst data suggest they could predict for greater sensitivity to PI3K pathway inhibition, not all sensitivity to PI3K inhibitors can be explained by these markers [2], [3], [4]. PI3K pathway activation has also been implicated in resistance to endocrine therapy in patients with estrogen receptor (ER) -positive breast cancers [5]. Recent results from the BOLERO-2 trial highlight that targeting mTOR is a viable strategy in patients with metastatic ER-positive disease who have progressed on previous endocrine therapy with a nonsteroidal aromatase inhibitor. The toxicity from the combination was not insignificant [6]. Hence, means to identify which ER-positive breast cancer patients may require PI3K/mTOR inhibition in addition to their endocrine therapy could facilitate better selection of patient populations for treatment, particularly in the adjuvant setting.

We have previously reported a gene signature (*PIK3CA*-GS) derived from patients with estrogen receptor (ER)-positive breast cancer with *PIK3CA* kinase domain mutations which was able to predict *PIK3CA* mutation status (genotype) in independent datasets and was also associated with better outcomes in tamoxifen-treated ER-positive breast cancers [7]. Several other investigators have also reported that the *PIK3CA* mutated compared with wild-type genotype is associated with a better prognosis, though a robust prognostic study is yet to be performed in large clinical breast cancer cohorts [7], [8], [9], [10]. Whilst the mechanism for this is unclear, at the gene expression level we observed unexpectedly low levels of PI3K/mTOR pathway activation and increased levels of estrogen signaling in *PIK3CA* mutant breast cancers. Hence, high levels of the *PIK3CA*-GS in estrogen receptor (ER)-positive breast cancer were associated with PIK3CA mutant (genotyped) breast cancers but paradoxically low mTORC1 pathway output. This led us to ask the question if the signature could provide an indication of pathway activation in ER-positive breast cancer patients, how this would compare to mutation status *per se* and importantly, if it could identify patients who may benefit from addition of a PI3K/mTOR pathway inhibitor as well as their hormonal therapy.

In this study we evaluated the ability of both *PIK3CA* genotype and the *PIK3CA*-GS to quantify the level of PI3K/AKT/mTOR pathway activation in a given tumor and its associations with anti-proliferative responses in ER-positive breast cancer treated with everolimus. The main endpoint of interest in this correlative analysis was centrally reviewed Ki67 immunohistochemistry (IHC) after two weeks of therapy. Both the change in percentage positively staining Ki67 measured from baseline to day 15 whilst on therapy, hereby referred to as “relative %Ki67 decrease” and the number of patients achieving a “complete cell cycle response” (a day 15 absolute Ki67 value of less than one after log transformation) were evaluated. Both of these endpoints have been shown to be correlated with long term outcomes in ER-positive breast cancer- ie have potential as early biomarkers of efficacy prediction of new drugs and have been used in clinical trials in this disease for this purpose [11], [12], [13], [14].

Importantly, one of the data sets examined in this study contained samples from a randomized clinical trial. This allowed us the opportunity to determine whether a predictive association existed between the *PIK3CA*-GS and the letrozole/everolimus combination compared with the letrozole alone. A biomarker that can identify individuals with a high chance of benefit from a drug may ultimately have more utility in the clinic.

## Materials and Methods

### Patient Populations

Two independent datasets (A and B) were used for these analyses and the patient populations have been previously described [14], [15], [16]. None of the described analyses were prospectively pre-planned in either study protocol.

Dataset A was a subset of patients from a randomized, double-blind, placebo-controlled, multi-centre, phase II neoadjuvant trial conducted in operable, previously untreated ER-postiive breast cancer greater than 2 cm at diagnosis in post-menopausal women [14]. Patients were randomly assigned to receive letrozole (2.5 mg/daily) plus placebo or letrozole plus the allosteric mTORC1 inhibitor everolimus (10 mg/daily), provided by Novartis Pharma AG (Basel, Switzerland), for 4 months prior to primary surgery. Of the original trial cohort, only 58 (21.5%) patients had pre-treatment (or baseline) samples profiled using Affymetrix U133A GeneChips™ and standard protocols. One patient was found to be ER-negative and removed from further analysis. No major differences between this subset and the global trial population in terms of clinical characteristics were observed (Table 1). This randomized dataset was used to examine the capabilities of PIK3CA genotype and the *PIK3CA*-mutant associated gene signature *(PIK3CA*-GS) to predict benefit from the addition of everolimus to endocrine therapy (letrozole).

**Table 1:** Baseline characteristics of the two datasets used in this analysis.

Variable	Dataset A (included in this analysis)	Dataset A Original phase II population#	Dataset B
**Mean age (years)**	**68.08yrs**	67.45 yrs	**68.1yrs**
SD	**9.05**	8.99	**10.1**
**Histologic Grade**			
I	**7 (12.1%)**	18 (6.7%)	**3 (13%)**
II	**38 (65.5%)**	105 (39%)	**10 (42.5%)**
III	**12 (20.6%)**	52 (19.2%)	**10 (42.5%)**
unknown	**1 (1.8%)**	95 (35%)	**NA**
**Clinical Tumor size at baseline**			
<2.0 cm	–	–	**5 (26.1%)**
>2.0 cm	**58 (100%)**	270 (100%)	**17 (73.9%)**
**Trial design**	Randomized Letrozole (2.5 mg/daily)+placebo OR; Letrozole+Everolimus (10 mg/day)		Single arm everolimus (5 mg/day)
**Total days of therapy**	**120**	120	**14**
**Total number of patients**	**58 (21.5% of original cohort)**	270	**23**
**Relative %Ki67 decreases** **(D0-D15)**	**85.6±4.1%(letrozole/everolimus)**	90.7% (letrozole/everolimus)	**30.9±13.9%**
	**60.8±8.55%(letrozole/placebo)**	74.8% (letrozole/placebo)	
**No. Absolute D15 Ki67 responders (defined as Ki67<1)** **	**18 (58% letrozole/everolimus)**	52 (57% letrozole/everolimus)	**3 (14%)**
	**6 (22% letrozole/placebo)**	25 (30% letrozole/placebo)	
**Frequency PIK3CA mutation**	**37.9% (22/58)**	35.8% (76/212)	**26% (6/23) $**
**Gene expression profiling**	Affymetrix GeneChips U133A As per standard protocols (Affymetrix, Santa Clara, CA).		Illumina HumanRef -8 v2 Expression Beadchip (Illumina, Cambridge, UK)

The second column compares the dataset A with the original previously published global trial population.

# as previously published in Baselga et al, JCO 2009 [11] (original cohort of which the Dataset A is a subset); Dataset B published by Sabine et al, BCR 2010 [13].

** As previously defined in Dowsett et al (CCR 2007) [9];

Dataset B was comprised of 23 post-menopausal patients also diagnosed with operable, previously untreated, ER-positive BC. These patients were enrolled in a single arm, single institution pre-surgical study to receive 5 mg of everolimus daily without therapeutic intent for 14 days prior to definitive surgery. All 23 patients had pre-treatment (baseline) tumor samples profiled using Illumina HumanRef-8 v2 Expression Bead-Chip (Illumina, Cambridge, United Kingdom). This dataset was used to examine the association between *PIK3CA* genotype and the *PIK3CA*-GS with changes in phosphorylated S6, a target of everolimus, as well as changes in Ki67, though it is unknown how changes in Ki67 induced by a mTOR inhibitor relate to prognosis in ER-positive BC.

### Endpoints

The main endpoints for this biomarker study were based on centrally reviewed proliferative measurements of Ki67 expression by immunohistochemistry (IHC) log transformed as previously described by Dowsett and colleagues [11], [12]. The endpoint of primary interest was the relative percentage change, as measured from baseline to day 15 post-treatment (i.e. relative %Ki67 decrease). Anti-proliferative response, as measured by reduction in Ki67 expression to a natural logarithm of percentage positive staining cells of less than 1 at day 15 (“absolute D15 Ki67 responders”) was also assessed. Both Ki67 endpoints have been shown to be predictive and prognostic in ER-positive BC treated with endocrine therapy [11], [12]. Hence, despite the difference in treatment duration, both data sets had endpoints measured by centrally reviewed Ki67 IHC at the same early time point (albeit a separate pathologist for each study).

In the original trial cohort, whilst changes in tumor size (complete and partial responses) assessed using clinical palpation were significantly different between the letrozole/everolimus vs letrozole/placebo arms (68.1% vs 59.1%, p = 0.0616) [14], in the cohort used for this study, we noted that the objective response rates were not significantly different (77.4% vs 66.7%, p = 0.323 respectively). Therefore, no associations between *PIK3CA* genotype or GS with objective tumor responses were performed.

### Changes in Phosphorylated S6 (S240) using IHC

Phosphorylated levels of S6 (S240) were also measured using IHC for dataset A and B. The methodology has been previously described [14],[15].

### Calculation of the *PIK3CA* Mutant-related Gene Signature (*PIK3CA*-GS)

Gene expression data were normalized and log-transformed prior to analysis. The *PIK3CA*-GS was calculated exactly as previously published and is essentially a weighted average of 278 probe sets using weights of +1 or −1, predefined by the association between the gene and the *PIK3CA* mutation status in the original training series [7]. For dataset B, all genes were matched to Entrez Gene IDs and mapped to Illumina probe sets prior to calculation of the signature score. Signatures scores and Ki67 information for both datasets are provided in Table S1. Note that high levels of the PIK3CA-GS scores represent low mTORC1 pathway activation.

### PIK3CA Mutation Analysis

PIK3CA mutation hot spot sequencing for dataset A and B has been previously described [14], [15]. For dataset A, there were 22 PIK3CA mutant (38.5%) and 35 PIK3CA WT tumors. For dataset B, there were 6 PIK3CA mutant (26%) and 17 PIK3CA WT. Analysis according to mutation location was not performed given the small number of mutated samples per dataset.

### Statistical Analyses

Several different analyses were planned for this biomarker study. First, because the *PIK3CA*-GS was developed by distinguishing *PIK3CA* genotyped (hot spot) mutant and WT tumors, we assessed the association between the *PIK3CA*-GS and *PIK3CA* genotype in these two datasets. The *PIK3CA*-GS score was used as a continuous variable. Receiver Operating Characteristic (ROC) curves were constructed and predictive ability was assessed by calculating the area under the curve (AUC) together with 95% confidence intervals (CI) using the trapezoidal rule.

Secondly, we investigated the association between relative %Ki67 change at day 15 from baseline and (a) *PIK3CA* gene status on its own, (b) the *PIK3CA*-GS alone and (c) a combination of the two variables. A linear regression approach was used for this purpose, with log transformed Ki67 values as dependent variable, and treatment arm, *PIK3CA* mutation status, the continuous *PIK3CA* gene signature as independent variables. When possible in the dataset A, responders were also defined by “absolute D15 Ki67” values as described above. A test for interaction was used to verify whether the slopes of the regression lines between *PIK3CA*-GS (or the coefficients of the *PIK3CA* mutation status) and relative change in Ki67 (log transformed) differed according to treatment arms. For visualization purposes, patients were ranked according to their *PIK3CA*-GS score and three groups of increasing values were obtained using the tertiles.

As an exploratory analysis, changes in percentage staining of phosphorylated S6, one of the substrates of mTOR (pS6 S240), as measured by IHC Histo-score between baseline and day 15 were correlated with *PIK3CA* genotype and *PIK3CA*-GS. For the regression line, log transformed values of pS6 were used.

Analyses were performed using SPSS version 20.0 (Chicago, IL). All p-values were two-sided and values ≤0.05 were considered significant. No correction for multiple hypotheses testing was taken as the results were considered hypothesis generating. The Reporting Recommendations for Tumor Marker Prognostic Studies (REMARK) criteria were followed in this study [17] however, the results and analyses presented in this study are considered exploratory and require further confirmation in other datasets.

## Results

### Patient Characteristics

Eighty-one patients were represented in the two clinical datasets A and B used to assess the association of *PIK3CA*-GS with mutation status and anti-proliferative response to letrozole with or without the mTOR inhibitor everolimus or everolimus alone. Baseline characteristics of these data sets are summarized in Table 1.

Concerning this study’s primary biological endpoint, relative %Ki67 decrease, dataset A showed 85.6±4.1% in the letrozole/everolimus compared with 60.8±8.55% in the letrozole/placebo arm (p = 0.001, Table 1). This is slightly lower but still comparable to the mean reduction of 90.7% vs. 74.8% (p = 0.0002) respectively reported in the full trial population [14]. In dataset B, in which patients had received everolimus alone at a lower dose, the relative %Ki67 decrease was much smaller, 30.9±13.9% [15], [16].

The number of absolute D15 Ki67 responders in dataset A was 20/31 (64.5%) in the letrozole/everolimus arm compared with 6/27 (22%) in the letrozole/placebo arm, again comparable to the global trial cohort. There were only three absolute D15 Ki67 responders in dataset B.

### Association between *PIK3CA*-GS and *PIK3CA* Genotype

**Figure 1:**
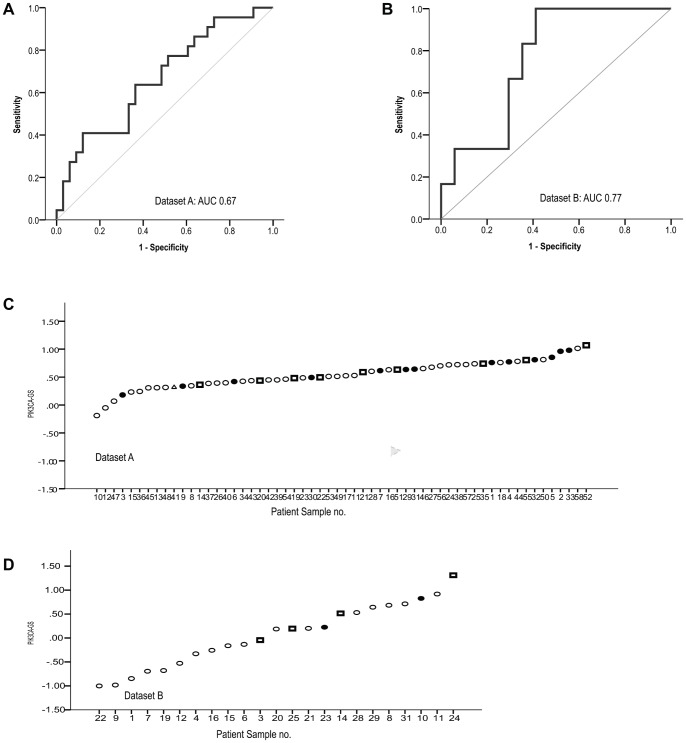
Receiver Operating Characteristic (ROC) curves showing the association of the *PIK3CA*-GS with *PIK3CA* mutation status. A. Dataset A, n = 22/58 (38.5%) AUC = 0.67, 95%CI: 0.5–0.8, p = 0.04), **B.** Dataset B n = 6/23 (26%) AUC 0.77, 95%CI : 0.6–0.96, p =  = 0.059). **C** and **D** show the individual samples for each dataset and distinguish between sequenced kinase (exon 20- squares) and helical (exon 9-circles) mutations and wild-type (WT-black) *PIK3CA*.

The incidence of *PIK3CA* mutations was 38.5% (22/58) in dataset A and 26% (6/23) in dataset B. The *PIK3CA*-GS was associated with *PIK3CA* mutation status in both datasets with ROC curves evaluating the *PIK3CA*-GS as a continuous variable (Figure 1A, 1B- dataset A: AUC = 0.67, 95% CI:0.52–0.81; p = 0.038; dataset B: 0.77, 95% CI:0.6–0.96; p = 0.059). Both kinase and helical domain mutations were detected with the signature (Figure 1C, 1D), with high levels of the GS, indicating closer similarity to the mutant genotype consistent with our previous study–ie signifying low levels of pathway activation [7].

### PIK3CA Mutation Genotype and Analysis of Anti-proliferative Response

Baseline Ki67 expression was not statistically different between *PIK3CA* mutant and WT BCs in both datasets. In dataset A, the mean expression was 31.82±5.17% and 36.58±3.8% (p = 0.34) compared with dataset B where the mean Ki67 was 14.08±3.14% and 21.68±3.08% (p = 0.45) in mutant and WT tumors respectively.

**Figure 2:**
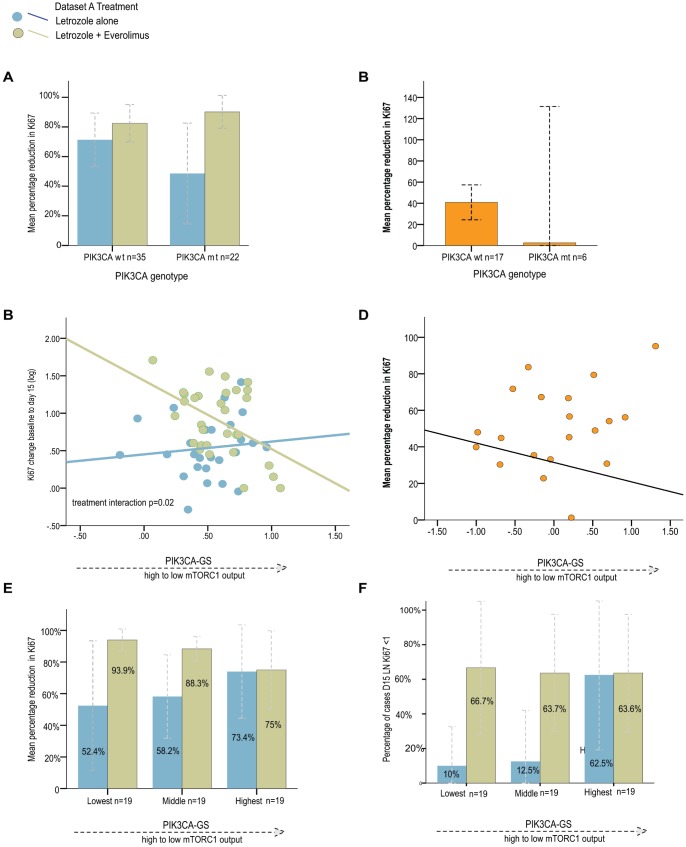
Relative change in %Ki67 from baseline to day 15 by treatment arm. According *PIK3CA* genotype in **A** dataset A; B dataset **B** According to *PIK3CA*-GS scores by treatment arm: regression lines for relative change in %Ki67 from baseline to day 15 **C** dataset A; **D** dataset B **E** Relative change in %Ki67 from baseline to day 15 by treatment arm and according to increasing tertiles of the *PIK3CA*-GS in dataset A; **F** Number of Absolute D15 responders by treatment arm and according to increasing tertiles of the *PIK3CA*-GS in dataset A.

The relationship between *PIK3CA* genotype and changes in %Ki67 decrease was investigated. In dataset A, no significant differences were observed in the letrozole alone arm between patients with PIK3CA mutant and WT BCs (mean %Ki67 decrease: 48.57±15.45% vs 71.3±8.3% respectively, p = 0.19, Figure 2A). The mean relative %Ki67 decrease in the letrozole/everolimus arm was similar for both PIK3CA mutant and WT BCs (82.4±6.03%. vs. 90.1±4.8% respectively, p = 0.42); there was no significant interaction between the two arms (p = 0.45; Figure 2A). In dataset B, the mean relative %Ki67 decrease to single agent everolimus was 2.61±50.17% vs 40.84±7.77% in patients with PIK3CA mutant and WT BCs respectively (p = 0.83) (Figure 2B).

### Association between *PIK3CA*-GS and Relative Anti-proliferative Responses in Dataset A and B

We investigated the correlations between relative %Ki67 changes and the *PIK3CA*-GS using a linear regression with Ki67 values (log transformed) as dependent variable, with treatment arm and the continuous *PIK3CA* GS as independent variables (Figure 2C). A test for interaction was used to verify whether the slope of the regression line differed between treatments. We found that *PIK3CA*-GS (as a continuous variable) and relative %Ki67 decreases were associated with a significant negative trend for the letrozole/everolimus arm (R = −0.43, p = 0.008), but not the letrozole/placebo arm (R = 0.07, p = 0.58) with a significant test for interaction between the arms (p = 0.02). In other words, lower *PIK3CA*-GS scores had the greatest reductions in %Ki67 in letrozole/everolimus treated patients, consistent with the concept that lower scores identified tumors with higher pathway activation.

Although Ki67 changes have shown to have prognostic value only for endocrine therapy, for completeness, we also evaluated the *PIK3CA*-GS in dataset B where single agent everolimus (5 mg/daily) was given [15], [16]. We observed no significant correlation between *PIK3CA*-GS and %Ki67 decreases in this dataset (R = −0.13, p = 0.37; Figure 2D).

For improved visualization, we compared the mean %Ki67 decreases in the letrozole/placebo and letrozole/everolimus arms by dividing the dataset A into three categories of increasing *PIK3CA*-GS values (Figure 2E). As seen, the Ki67 decreases were the largest in the combination treatment arm in patients with the lowest *PIK3CA*-GS scores. We also performed the association analysis between the *PIK3CA*-GS and the endpoint of achieving an absolute D15 Ki67 response in dataset A, even though the number of these responders were small, the results were similar (Figure 2F; interaction p test = 0.17). In dataset B, given the small numbers no further analyses were performed.

### Combination PIK3CA-GS, PIK3CA Genotype and Anti-proliferative Response

**Figure 3:**
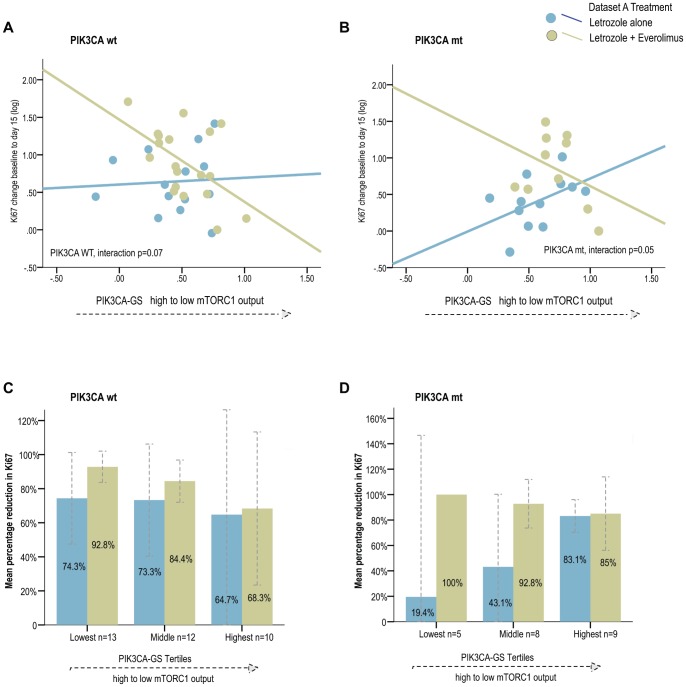
Regression lines for relative change in %Ki67 from baseline to day 15 according to *PIK3CA*-GS scores by treatment arm by *PIK3CA* genotype in dataset A. A PIK3CA WT; **B** PIK3CA mutant Relative change in %Ki67 from baseline to day 15 by treatment arm and according to increasing tertiles of the *PIK3CA*-GS by *PIK3CA* genotype in dataset A **C** PIK3CA WT; **D** PIK3CA mutant.

It was relevant then to examine the anti-proliferative response of the *PIK3CA*-GS in each *PIK3CA* genotype to determine if a combination of genotype and phenotype could provide improved discrimination of responders. The results from dataset A suggest that the *PIK3CA*-GS performs similarly for both *PIK3CA* genotypes (Figure 3). Therefore, the data suggest that even for patients with a *PIK3CA* mutation there is heterogeneity in pathway signaling - in other words, those *PIK3CA* mt tumors with the lowest *PIK3CA*-GS scores (high pathway output) were associated with larger reductions in Ki67 with letrozole/everolimus compared with letrozole alone (correlations as a continuous variable: letrozole/everolimus R = −0.36, letrozole/placebo R = 0.47; interaction test p = 0.05, Figure 3A, 3B). This pattern was similar for the *PIK3CA* WT genotype (letrozole/everolimus R = −0.54, letrozole/placebo R = 0.06; interaction test p = 0.07). Again, for improved visualization, tertiles of the *PIK3CA*-GS signature score were used for both mutant and WT population (Figure 3C, 3D). This demonstrates that lower levels of the *PIK3CA*-GS was associated with larger Ki67 differences according to treatment arm- in other words, better responses to letrozole/everolimus were observed compared with letrozole/placebo consistent with increased pathway activation.

In dataset B, given the small number of *PIK3CA* mutated BCs this analysis was not performed.

### Association between PIK3CA-GS and Relative Change in Phosphorlyated-S6 (S240)

**Figure 4:**
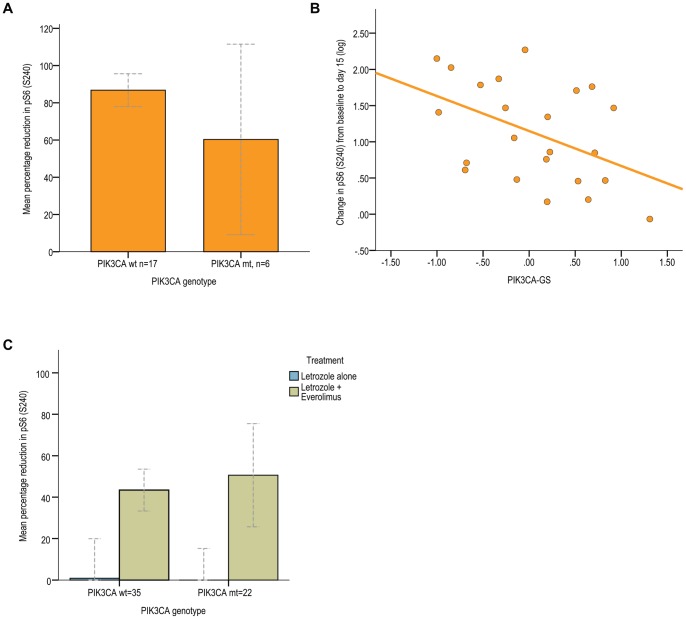
Associations between PIK3CA-GS and relative change in phosphorylated S6. A Dataset B: relative percentage change in pS6 (S240) as measured by IHC from baseline to day 15 according *PIK3CA* genotype **B** Regression line for relative change in pS6 from baseline to day 15 according to *PIK3CA*-GS in dataset B **C** Dataset A: Relative percentage change in phosphorylated S6 (S240) as measured by IHC from baseline to day 15 according *PIK3CA* genotype and treatment arm.

Given that anti-proliferative responses using KI67 were developed for ER-positive BC treated with endocrine agents, we looked at the relationship between the *PIK3CA*-GS, *PIK3CA* genotype and changes in phosphorylated S6 (pS6 S240) detected using IHC in the pre-surgical study cohort of dataset B (everolimus alone). The mean change in percentage positive cells staining for pS6 from baseline to day 15 for all patients was 79.9±6.24%. As seen, there was no significant difference seen for percentage change in pS6 according to genotype (p = 0.45, Figure 4A). In contrast, there was a significant inverse correlation seen between the *PIK3CA*-GS and changes in pS6 (R = −0.46, p = 0.028 Figure 4B). Of note, there was no association between changes in pS6 and changes in Ki67 (R = 0.09) in dataset B.

In dataset A, interestingly for the combination, the relative decrease observed for pS6 was less: the mean change was 47.4±4.7% in the letrozole/everolimus arm. As expected, there was minimal decrease for letrozole alone: 0.33±5.7%. There was no significant difference in the relative change in pS6 according to PIK3CA genotype (p = 0.51, Figure 4C). There was no significant correlation between relative pS6 changes and PIK3CA-GS as a continuous variable (R = 0.11). Of note, there was no association between pS6 and the main endpoint, %Ki67 changes (R = 0.008), in the letrozole/everolimus arm.

## Discussion

The efforts invested into developing therapeutics targeted against the PI3K signaling pathway have been immense due to the knowledge that deregulation of this pathway contributes to increased tumor growth and proliferation [18]. Equally, considerable attention has been devoted to the search for molecular predictive biomarkers of response to PI3K pathway inhibitors [3], [19]. Particularly for the ER-positive or “luminal” BCs, *PIK3CA* mutations occur commonly and are thought to represent a logical target population for this therapy. However, several issues have complicated *PIK3CA* mutations as a predictive biomarker in BC: (i) *PIK3CA* mutations have been associated with a relatively better prognosis compared with *PIK3CA* wild-type BC patients [7], [9], (ii) the mutations are not associated with higher proliferation indices or lower efficacy with hormonal therapy which would be hypothesized from oncogenic activation of *PIK3CA* [8], and (iii) *PIK3CA* mutant breast cell lines have been associated with sensitivity to tamoxifen [7], [20]. Hence the effect of *PIK3CA* mutational activation on signaling and clinical relevance in ER-positive BC is unclear.

Our results, whilst derived from two small studies have important conclusions. These data support our original observations the high levels of the *PIK3CA*-GS is associated with the *PIK3CA* mutant genotype. However, here we provide further data to support that higher values of the *PIK3CA*-GS (i.e.: the mutant-like phenotype) are associated with low levels of pathway activation [7]. Whilst this may seem counterintuitive, this could be related to the fact that the molecular composition of the tumors defined as *PIK3CA* wild-type is unknown- in other words, there are probably multiple other means of PI3K pathway activation that exist in this group– or that low as opposed to high levels of pathway activation could be enough in the luminal breast cancer subtype for reasons yet to be fully understood- ie for example, too high levels may result in senescence. Furthermore, in this manuscript we report that those ER-positive BCs with lower *PIK3CA*-GS scores and therefore higher levels pathway activation had greater %Ki67 reductions with the combination letrozole/everolimus treatment compared with letrozole alone–in other words, a significant interaction is seen between treatment arms. Importantly in dataset A the early Ki67 changes were reported to correspond with the better objective responses observed with the combination arm letrozole/everolimus arm compared with letrozole alone, supporting its use as a potential prognostic marker and surrogate endpoint for neoadjuvant trials in endocrine therapy and highlighting the potential significance of our findings [11], [12], [13].

Admittedly, the results from the second dataset of single agent everolimus alone are hard to interpret given that prognostic value of early Ki67 changes have only been shown for endocrine therapy and no clinical outcome data is currently available (ie no tumor shrinkage or relapse information). As dataset B was designed to be a pre-surgical study, it can only be concluded that everolimus was indeed “hitting its target” in terms of S6 reductions and the *PIK3CA*-GS (but not genotype) was correlated with these changes. In contrast, the *PIK3CA*-GS (and not genotype) was associated with Ki67 responses but not changes in pS6 in dataset A. Dataset A involved a randomization between endocrine therapy with and without everolimus. In dataset A, changes in Ki67 reflected the primary endpoint results (objective decrease in tumor size), whereas changes in phosphorylated S6 were not correlated with clinical outcomes and seemed to indicate a purely pharmacodynamic effect [14]. Several hypotheses are possible for why changes in Ki67 and pS6 had different implications in these two datasets: (i) the PIK3CA-GS is not specific to everolimus alone, but could incorporate a measurement of estrogen signaling given that the PIK3CA-GS was developed from ER-positive, *PIK3CA* mutant BC samples– many ER-related genes are present in the gene signature and this could explain why the signature could be conceivably also affected by endocrine therapy and have correlations with anti-proliferative Ki67 reductions in this context (ii) the small sample size in dataset B limited our power to see subtle Ki67 effects with the single agent everolimus, (iii) the combination of letrozole/everolimus altered the effects on pS6 reductions compared with everolimus alone, (iv) different patient populations between datasets (v) the lower dose of everolimus and finally (vi) technical issues could all be contributing factors to these observations [21]. Whilst further study is required to clarify the relationship between the signature, different biomarkers and single vs combination therapy, for this study, dataset A probably provides the most persuasive data due to the randomization and some clinical outcome information being available.

Importantly, whilst we cannot exclude the possibility that *PIK3CA* mutation status alone could yield the same prognostic or predictive information as the *PIK3CA*-GS in larger clinical trial data sets that would have more statistical power, notably there was no interaction seen previously in the whole trial cohort of dataset A involving nearly 300 women [14]. Larger studies will also be able to analyze the effects of *PIK3CA* mutation status and *PIK3CA*-GS on more robust clinical endpoints such as survival other than Ki67 changes. It is also possible that *PIK3CA* genotype may be more predictive of sensitivity to specific PI3K or AKT inhibitors rather than mTOR. Regardless, future phase II/III trials should consider upfront stratification by *PIK3CA* genotype in order to accurately determine efficacy in this population, especially given its high frequency. Perhaps the combination of mutation status (genotype) and *PIK3CA*-GS (phenotype) could allow optimal therapy individualization for this particular aberration. Moreover, PIK3CA genotype may have different implications in early vs. metastatic settings,

HER2 negative vs. overpressing breast cancer subtypes – the latter could imply further dependency on p110α or higher “oncogene addiction” to PI3K signaling.

We acknowledge that none of these analyses were prospectively pre-planned in the study protocols and the cohort sizes were small so the conclusions are hypothesis generating. Nevertheless, the strengths of our analysis derive from the fact that the validation was performed using data from prospectively designed clinical trials - one of which was randomized. Importantly, both trials used the same centrally reviewed Ki67 endpoint. Notably, these results highlight the importance of prospective tumor banking, the use of standard endpoints in clinical trials and collaboration between academia and pharma in order to facilitate biomarker validation. Without concerted efforts in this regard, the hope for individualized cancer treatment will remain elusive. As seen, we could only evaluate the *PIK3CA*-GS in two small cohorts, as correlative tumor collection is not standard in many clinical studies. Yet, this is the first time a PI3K pathway-related microarray-derived gene signature has been validated in an independent randomized, prospective clinical trial dataset for its potential as a predictive biomarker to PI3K-pathway targeted agents.

In conclusion, whilst it is clear that many ER-positive BC patients do well with endocrine therapy alone [14], [22], recent clinical trial data presented in the ER-positive metastatic BC setting provide substantial support for mTOR pathway activation as a major means of estrogen independent tumor growth in this population [6], [23]. Hence, the ability to identify which ER-positive patients could benefit most from the combination in newly diagnosed tumors is now crucial to understanding who needs this combination in the adjuvant setting. Our study supports further investigation of the *PIK3CA*-GS in appropriate randomized datasets as a biomarker that could identify women who may derive benefit from the addition of everolimus to letrozole in ER-positive disease.

### Access to Gene Expression Data

De-identified gene expression data for dataset A is available on request to David Chen, Novartis David.chen@novartis.com. Gene expression data for dataset B is available at https://catissuesuite.ecmc.ed.ac.uk/caArray. We acknowledge the Edinburgh Cancer Medicine Centre and Cancer Research UK for tissue banking support for dataset B.

## Supporting Information

Table S1 **Table gives** ***PIK3CA*** **-GS scores and clinical information for both datasets.**
(XLS)
