# Correction: PIK3CA Genotype and a PIK3CA Mutation-Related Gene Signature and Response to Everolimus and Letrozole in Estrogen Receptor Positive Breast Cancer

**DOI:** 10.1371/journal.pone.0216175

**Published:** 2019-04-23

**Authors:** Sherene Loi, Stefan Michiels, Jose Baselga, John M. S. Bartlett, Sandeep K. Singhal, Vicky S. Sabine, Andrew H. Sims, Tarek Sahmoud, J. Michael Dixon, Martine J. Piccart, Christos Sotiriou

The following information is missing from the Competing Interests statement: JB is a consultant to Novartis, Roche, Merck, Sanofi-Aventis, Verastem, Bayer, Chugai, Exelixis, Onyx and Constellation.
